# Predictive value of chest HRCT for survival in idiopathic pulmonary arterial hypertension

**DOI:** 10.1186/s12931-021-01893-8

**Published:** 2021-11-17

**Authors:** Aneta Kacprzak, Barbara Burakowska, Marcin Kurzyna, Anna Fijałkowska, Michał Florczyk, Maria Wieteska-Miłek, Szymon Darocha, Adam Torbicki, Monika Szturmowicz

**Affiliations:** 1 1st Department of Lung Diseases, National Tuberculosis and Lung Diseases Institute, Płocka 26, 01-138 Warsaw, Poland; 2 Radiology Department, National Tuberculosis and Lung Diseases Institute, Warsaw, Poland; 3 Department of Pulmonary Circulation and Thromboembolic Diseases, Medical Centre of Postgraduate Education, European Health Centre Otwock, Otwock, Poland; 4 Department of Cardiology, Institute of Mother and Child, Warsaw, Poland

**Keywords:** Idiopathic pulmonary arterial hypertension, High resolution computed tomography, Ground-glass opacifications, Survival, Risk factors

## Abstract

**Background:**

Little attention has been paid to chest high resolution computed tomography (HRCT) findings in idiopathic pulmonary arterial hypertension (IPAH) patients so far, while a couple of small studies suggested that presence of centrilobular ground-glass opacifications (GGO) on lung scans could have a significant negative prognostic value. Therefore, the aims of the present study were: to assess frequency and clinical significance of GGO in IPAH, and to verify if it carries an add-on prognostic value in reference to multidimensional risk assessment tool recommended by the 2015 European pulmonary hypertension guidelines.

**Methods:**

Chest HRCT scans of 110 IPAH patients were retrospectively analysed. Patients were divided into three groups: with panlobular (p)GGO, centrilobular (c)GGO, and normal lung pattern. Association of different GGO patterns with demographic, functional, haemodynamic, and biochemical parameters was tested. Survival analysis was also performed.

**Results:**

GGO were found in 46% of the IPAH patients: pGGO in 24% and cGGO in 22%. Independent predictors of pGGO were: positive history of haemoptysis, higher number of low-risk factors, and lower cardiac output. Independent predictors of cGGO were: positive history of haemoptysis, younger age, higher right atrial pressure, and higher mixed venous blood oxygen saturation. CGGO had a negative prognostic value for outcome in a 2-year perspective. This effect was not seen in the longer term, probably due to short survival of cGGO patients.

**Conclusions:**

Lung HRCT carries a significant independent prognostic information in IPAH, and in patients with cGGO present on the scans an early referral to lung transplantation centres should be considered.

## Introduction

Pulmonary hypertension (PH) is defined as an increase in mean pulmonary artery pressure (PAPm) ≥ 25 mmHg at rest as assessed by right heart catheterisation (RHC) [1]; recently threshold of 20 mmHg has been proposed [2]. There are multiple clinical conditions associated with PH, and the classification of PH was created based on similarities in their pathophysiological mechanisms, clinical presentation, haemodynamic characteristics, and therapeutic management [1, 2]. There are five main groups of PH: 1. pulmonary arterial hypertension (PAH), 2. PH due to left heart diseases, 3. PH due to lung diseases and/or hypoxia, 4. PH due to pulmonary artery obstructions, 5. PH with unclear and/or multifactorial mechanisms. Each group is further divided into subgroups [1, 2]. Group 1, i.e. PAH, results primarily from pulmonary vascular disease involving pulmonary arteries, arterioles, capillaries and small veins. Vasoconstriction and remodelling of pulmonary vessels cause rise in pulmonary vascular resistance (PVR), which in turn leads to increase in PAP, and finally adversely affects performance and structure of the right heart ventricle causing its failure [1]. PAH belongs to pre-capillary PH category and haemodynamic definition corresponding with it includes: PAPm ≥ 25 mmHg, pulmonary artery wedge pressure (PAWP) ≤ 15 mmHg, PVR ≥ 3 Wood Units [1]. PAH group encompasses following subtypes: idiopathic (I)PAH, heritable (H)PAH, drug- and toxin-induced PAH, PAH associated with: connective tissue disease, human immunodeficiency virus (HIV) infections, portal hypertension, congenital heart disease, or schistosomiasis, PAH long-term responders to calcium channel blockers, PAH with overt features of venous/capillaries involvement, and finally persistent PH of the newborn syndrome [1, 2]. IPAH is diagnosed in cases without any family history of PH or known triggering factor [1]. HPAH occurs in a familial context due to mutations in PAH predisposing genes, most frequently in bone morphogenetic protein receptor 2 (BMPR2) gene [1]. There are no significant differences between IPAH and HPAH in terms of clinical, radiological, and haemodynamic presentation, the disease course, management or prognosis. PAH with overt features of venous/capillaries involvement includes pulmonary veno-occlusive disease (PVOD) and pulmonary capillary haemangiomatosis (PCH). In PVOD/PCH venous and capillary involvement is more prominent than in other PAH forms. PVOD/PCH may be idiopathic, heritable, induced by drugs, toxins, and radiation, or it can complicate the course of connective tissue disease and HIV infection [1]. More pronounced pulmonary venous/capillary involvement is associated with a poor prognosis, a limited response to PAH therapy and a risk of pulmonary oedema with these treatments [1, 3–5]. Ultimate distinction between PVOD/PCH and I/HPAH can be made only on histopathologic or genetic basis [1]. The former can be applied only either post-mortem or in cases of lung transplantation (LTx) because lung biopsy is contraindicated in PH. The latter requires confirmation of biallelic mutations in the eukaryotic translation initiation factor 2alpha kinase 4 (EIF1AK4) gene — present in all patients with heritable form of PVOD/PCH and in about 25% of sporadic cases [1, 5]. However, the diagnosis may be strongly suspected in PAH cases with very low lung diffusion capacity for carbon monoxide (DLCO), resting hypoxemia, severe exertional desaturation, pulmonary oedema in response to PAH therapy, and the characteristic triad of findings on chest high resolution computed tomography (HRCT): centrilobular ground-glass opacities, mediastinal lymphadenopathy, and smooth thickening of the interlobular septa [1, 3–6]. Normal chest HRCT doesn’t exclude PVOD/PCH, though [3, 5].

Ground-glass opacities (GGO) is a radiological term for areas of hazy increased lung parenchyma density not sufficient to obscure bronchial and vascular margins on chest HRCT [7]. They are non-specific findings, and there are two patterns of GGO distribution: centrilobular (cGGO) and panlobular (pGGO) [7, 8]. CGGO, also called centrilobular nodules, are related to centrilobular core structures such as arterioles, bronchioles, a surrounding lymphatic network, and supporting connective tissue. On HRCT scans centrilobular nodules may have focal, multifocal, or diffuse appearance. CGGO correspond to various types of histological pictures depending on disease entity [8]. PGGO usually reflect affection of the air space, interstitium, or both these compartments; it can be caused by alveolar or interstitial oedema, an inflammatory process or alveolar haemorrhage [7–9]. In IPAH, lung scans are expected to display no significant abnormalities that would suggest underling respiratory disease [1]. On the other hand, it is now well recognised that up to 50% of PAH patients may present GGO on HRCT scans despite not having any airway or lung parenchymal disease [1, 3, 6, 10–17]. CGGO were seen in 23–28% of patients with I/H/anorexigen-induced PAH, with no signs of PVOD/PCH on lung pathologic evaluation [3, 6, 15].

IPAH remains a life-threatening disease despite constant development of therapeutic options and improved management plans [1, 18–24]. Current medical therapies aim to overcome imbalance between vasoactive and vasodilator mediators and restore the endothelial cell function in pulmonary arterial bed, as these elements play essential role in pulmonary vascular remodelling in PAH. PAH-specific medications include: prostacyclin analogues and receptor agonists, phosphodiesterase 5 inhibitors, endothelin-receptor antagonists, and cyclic guanosine monophosphate activators [1, 25]. The ultimate cure for advanced IPAH is LTx [1], but the procedure carries a significant risk of severe complications and death [26]. Therefore, a referral for LTx requires a careful consideration of risks and benefits. Referral timing should be based on a patient’s individual survival prognosis and expected waiting time on local transplant list [1, 27, 28]. There are few risk stratification tools, such as REVEAL score [29] and European Society of Cardiology (ESC)/European Respiratory Society (ERS) strategy [1], recommended for prognosis assessment [1, 28]. Still, many IPAH patients die being referred for LTx too late or not at all [18–23, 30, 31]. Neither REVEAL score nor ESC/ERS risk assessment tool incorporates findings of chest HRCT, while presence of cGGO on the lung scans has been reported to have a significant prognostic value for outcome in IPAH patients in few studies [11, 12].

Although well appreciated, the phenomenon of inhomogeneous lung attenuation in IPAH has not been thoroughly investigated and described. Consequently, the objectives of our study were: to assess frequency and clinical significance of GGO in IPAH, and to verify if it carries an add-on prognostic value in reference to ESC/ERS multidimensional risk assessment tool.

## Method

Patients diagnosed with IPAH in our hospital between 1997 and 2011, and for whom chest HRCT scans were available for review, were retrospectively analysed. Diagnosis of IPAH was based on evidence of PAH on RHC and exclusion of known causes of PH. All included patients had PAPm ≥ 25 mmHg, PAWP ≤ 15 mmHg, and PVR > 3 Wood units [1]. All patients underwent broad differential workup of PH. It included anamnesis covering personal and familial medical history, exposure to drugs and toxins, as well as physical examination findings and additional tests. Left heart diseases and congenital heart diseases were excluded with means of echocardiography and RHC (normal PAWP), lung diseases were excluded on the basis of pulmonary function testing and HRCT scanning, pulmonary artery obstruction was excluded with pulmonary artery contrast enhanced CT and perfusion lung scintigraphy. All patients had connective tissue disease ruled out, negative testing for HIV infection, and negative abdominal ultrasound for portal hypertension. Two of included patients had unclear familial history of PH. The studied group did not contain patients with high clinical suspicion of PVOD/PCH, i.e. patients with a characteristic triad of chest HRCT findings, or patients who developed pulmonary congestion in response to vasodilators. However, no histopathologic verification or genetic testing were performed, so the cohort could include patients with HPAH or PAH in the course of PVOD/PCH.

The HRCT examinations were performed between Jan 1997 and Nov 2011, with use of either a single-row helical unit Picker PQ 2000 (Siemens) or a multi-row Somatom Sensation 16 (Siemens). In part of the patients images were obtained with a sequential acquisition technique with 1-mm section thickness at 10 mm intervals, in others high resolution images were reconstructed from contrast enhanced spiral acquisition. All scans were acquired in the supine position, at end-inspiration. There were no differences in the radiological protocols between studies obtained with both CT equipments, and differences in technical specifications of both units made no impact on assessment of elements included in the analysis. The images were reassessed by the radiologist experienced both in PH and interstitial lung diseases, blinded to patients’ clinical details and outcomes. The main focus was on assessment of lung parenchyma homogeneity, and patients were divided into three groups: with cGGO pattern, with pGGO pattern, and with normal pattern. CGGO were further evaluated as distributed evenly or with a zonal predominance, pGGO as patchy or perihilar. Mediastinal lymphadenopathy (smallest diameter > 10 mm) and smooth thickening of interlobular septa were also recorded.

Data collected for analyses were: sex, age at the time of chest HRCT, cigarette smoking status, history of haemoptysis, presence of patent foramen ovale, World Health Organisation functional class (WHO FC), 6-min walking test results (distance, pre-test and the lowest exertional oxygen saturation measured with pulse oximeter), plasma N-terminal pro-brain natriuretic peptide (NT-proBNP), DLCO percent of predicted value (%pred), haemodynamic parameters: mean right atrial pressure (RAPm), systolic, diastolic and mean pulmonary artery pressures, cardiac output (CO) and cardiac index (CI), mixed venous blood oxygen saturation (Svo_2_), PVR [32], result of acute vasoreactivity testing (responder or non-responder according to ESC/ERS guidelines definition [1]). Median (IQR) interval between chest HRCT and RHC was 10 (3–74) days. All other risk factors were assessed simultaneously, i.e. during the same hospitalisation, with chest HRCT scanning.

For risk stratification 6 of 13 risk factors recommended in the 2015 ESC/ERS PH guidelines were used: WHO FC, 6-min walking test distance (6MWD), NT-proBNP plasma level, RAPm, CI, and SvO_2_, with cut-off values and three levels of risk as proposed in the document [1]. The method to calculate each patient’s risk was adopted from Kylhammar et al. [22]. The French approach based on number of low-risk factors was also applied [21].

The STATISTICA v. 13 (Statsoft) computer software was used for statistical analysis. Continues data were presented as median with interquartile range, and Kruskal–Wallis’ or U Mann–Whitney’s tests were used for comparison between three or two groups, respectively. Categorical variables were shown as actual number with percentage where appropriate, and the Pearson’s chi-square test was used for comparison between groups. Logistic regression was performed to determine factors associated with different HRCT patterns, the normal pattern was a reference class. The Somers’ D was used to built the multivariate regression models. Transplant-free survival was calculated from the date of chest HRCT scanning to the date of death or LTx (complete observation) or the date of last follow-up, with 23rd Apr 2020 terminating the period of observation. Survival analysis was performed using the Kaplan–Meier method. Survival between groups was compared with log-rank test. Univariable and multivariable Cox proportional hazards models were used to evaluate the predictive value of the HRCT patterns as well as the other analysed variables. Parameters with p < 0.2 in the univariate analysis were included in the multivariate analysis. Hazard ratios (HR) with 95% confidence interval (95% CI) were presented. P < 0.05 was considered statistically significant.

No ethics committee approval or informed consent were required due to retrospective character of this study. It was conducted in agreement with the Declaration of Helsinki and the European General Data Protection Regulation.

## Results

### General characteristics and chest HRCT findings

**Fig. 1:**
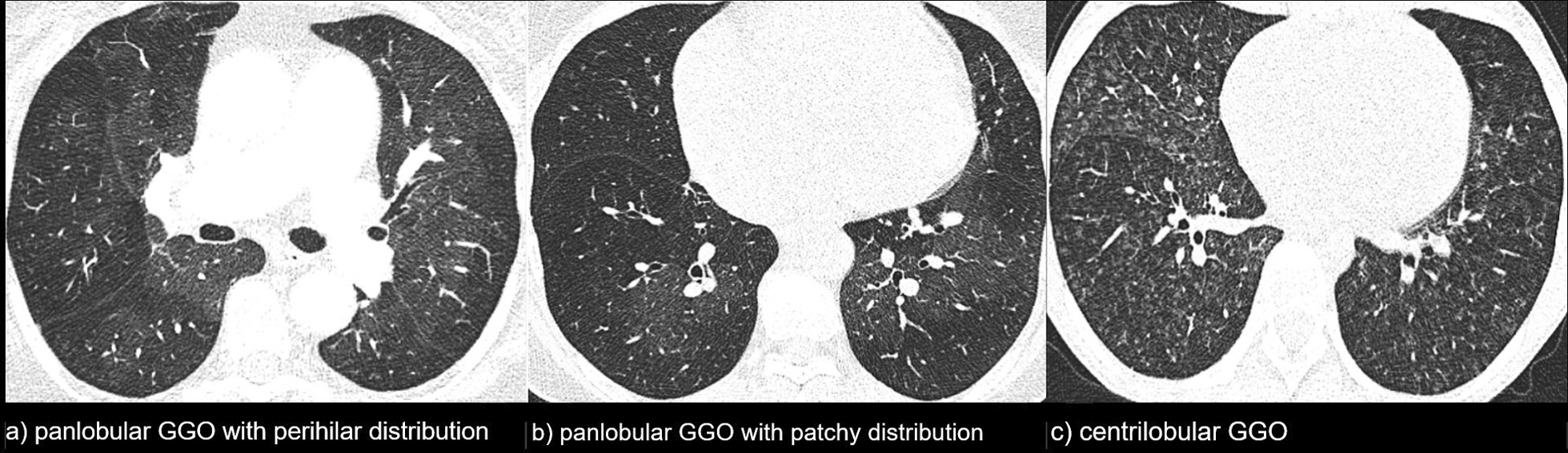
Chest HRCT scans of IPAH patients showing different patterns of ground-glass opacifications (GGO)

The studied group consisted of 110 IPAH patients: 102 (93%) incident, 80 (73%) women, age median value was 44.3 years (range 17.3–78.5 years). Fifty (46%) patients had the abnormal lung pattern on HRCT scans. The pGGO were found in 26 (24%) patients, perihilar localisation was seen in 50% of them. The cGGO were found in 24 (22%) patients, nodules were widespread and with no specific zonal predominance in all cases. The remaining 54% of the patients were classified as normal HRCT pattern. One patient with pGGO had lymphadenopathy and septal thickening. Among patients with cGGO, two had lymphadenopathy, and another one had septal thickening. Three patients with normal pattern had lymphadenopathy. The characteristics of the whole studied group, as well as detailed comparison of the three groups according to the lung HRCT pattern are presented in Tables 1 and 2, GGO patterns are shown in Fig. 1.

**Table 1:** Demographic, functional and biochemical parameters for the whole group and according to the HRCT pattern

	All patients		cGGO pattern		pGGO pattern		Normal pattern		p value*
	N	Median (IQR) or N (%)	N	Median (IQR) or N (%)	N	Median (IQR) or N (%)	N	Median (IQR) or N (%)	
Age [years]	110	44.3 (30.0–56.6)	24	32.1 (24.3–49.0)	26	44 (27–49.6)	60	46.8 (37.6–59.0)	0.064
Age < 40 years	110	44 (40)	**24**	**15 (63)**	**26**	**12 (46)**	**60**	**17 (28)**	**0.012**
Sex female:male	110	80:30 (73:27)	**24**	**20:4 (83:17)**	**26**	**23:3 (88:12)**	**60**	**37:23 (62:38)**	**0.016**
Ever-smokers	106	41 (39)	21	9 (43)	26	5 (19)	59	27 (46)	0.062
Positive history of haemoptysis	108	18 (16)	**24**	**7 (29)**	**26**	**6 (23)**	**58**	**5 (9)**	**0.046**
PFO	93	29 (31)	22	7 (32)	20	8 (40)	59	14 (27)	0.59
WHO FC:	107		24		26		57		0.14
1		0		0		0		0	
2		57 (53)		14 (58)		17 (65)		26 (46)	
3		44 (41)		7 (29)		8 (31)		29 (51)	
4		6 (6)		3 (13)		1 (4)		2 (4)	
DLCO [%pred]	84	66.8 (52.8–76.5)	15	58.7 (33.4–71.6)	21	70.6 (60.4–73.6)	48	66.7 (39.0–83.1)	0.27
6MWD [m]	105	396 (318–474)	21	436 (380–484)	26	415.5 (380–484)	58	379.5 (298.0–450.0)	0.06
6MWD risk group:	105		21		26		58		0.34
Low		37 (35)		10 (48)		11 (42)		16 (28)	
Intermediate		63 (60)		11 (52)		14 (54)		38 (66)	
High		5 (5)		0 (0)		1 (4)		4 (7)	
SpO_2_ [%]	105	86 (92–97)	21	96 (92–97)	26	96.5 (93–98)	58	95.0 (91.0–97.0)	0.20
6MWT ΔSpO_2_	102	5 (1–8)	21	5 (3–9)	26	4.5 (2–8)	55	4.0 (0.0–9.0)	0.50
NT-proBNP [pg/ml]	83	1079 (332–2592)	22	1619.5 (422.9–4331.0)	21	825 (219.9–1610.0)	40	1121.0 (360.0–2599.0)	0.30
NT-proBNP risk group:	83		22		21		40		0.54
Low		18 (22)		4 (18)		6 (28.5)		8 (20)	
Intermediate		29 (35)		6 (27)		9 (43)		14 (35)	
High		36 (43)		12 (55)		6 (28.5)		18 (45)	

Bold values denote statistical significance at the *p* < 0.05 level

cGGO, centrilobular ground glass opacifications; pGGO, panlobular ground glass opacification; IQR, interquartile range; PFO, persistent foramen ovale; WHO FC, World Health Organisation functional class; DLCO, diffusion lung capacity for carbon monoxide; 6MWD, 6-min walking test distance; SpO_2_, blood oxygen saturation; NT, proBNP- N-terminal pro-brain natriuretic peptide. *For comparison between three HRCT patterns

**Table 2:** Haemodynamic parameters and risk factors for the whole group and according to the HRCT pattern

	All patients		cGGO pattern		pGGO pattern		Normal pattern		p value*
	N	Median (IQR) or N (%)	N	Median (IQR) or N (%)	N	Median (IQR) or N (%)	N	Median (IQR) or N (%)	
RAPm [mmHg]	107	8 (5–11)	**24**	**10 (8–13)**	**26**	**7 (4–10)**	**57**	**8.0 (5.0–10.0)**	**0.024**
RAPm risk group:	107		24		26		57		0.055
Low		51 (48)		6 (25)		17 (65)		28 (49)	
Intermediate		44 (41)		13 (54)		8 (31)		23 (40)	
High		12 (11)		5 (21)		1 (4)		6 (11)	
PAPs [mmHg]	108	83 (69–97)	24	86 (69,5–102.5)	26	85 (73–95)	58	79.5 (67.0–93.0)	0.24
PAPd [mmHg]	108	39.5 (31–50)	24	43.5 (33.5–54.0)	26	40.5 (29–49)	58	37.5 (29.0–47.0)	0.15
PAPm [mmHg]	108	55 (46.5–67.5)	24	61.5 (48.0–73.5)	26	58.5 (46–67)	58	54.0 (44.0–64.0)	0.12
CO [l/min]	108	4.4 (3.7–5.2)	24	4.2 (3.6–5.1)	26	4.2 (3.7–4.6)	58	4.7 (3.7–5.3)	0.20
CI [l/min/m^2^]	108	2.5 (2.2–3.0)	24	2.5 (2.3–3.1)	26	2.6 (2.2–2.8)	58	2.5 (2.2–3.1)	0.90
CI risk group:	108		24		26		58		0.56
Low		54 (50)		10 (42)		16 (62)		28 (48)	
Intermediate		42 (39)		12 (50)		7 (27)	8	23 (40)	
High		12 (11)		2 (8)		3 (11)		7 (12)	
Svo_2_ [%]	108	61 (53.5–69.0)	24	63 (55.5–70.0)	26	61.5 (53–69)	58	59.0 (54.0–68.0)	0.68
Svo_2_ risk group:	108		24		26		58		0.32
Low		36 (33)		9 (38)		10 (39)		17 (29)	
Intermediate		23 (21)		8 (33)		4 (15)		11 (19)	
High		49 (45)		7 (29)		12 (46)		30 (52)	
PVR [Wood units]	108	10.3 (8.1–14.4)	24	12.6 (8.8–16.8)	26	9.9 (8.4–15.2)	58	9.8 (7.5–13.2)	0.15
Responder	102	22 (22)	23	5 (22)	23	7 (30)	56	10 (18)	0.47
Risk group:	110		24		26		60		0.57
Low		30 (27)		5 (21)		8 (31)		17 (28)	
Intermediate		66 (60)		15 (62)		17 (65)		34 (57)	
High		14 (13)		4 (17)		1 (4)		9 (15)	
Number of low-risk factors	110	2.0 (1.0–4.0)	24	2.0 (1.0–3.0)	26	3.0 (2.0–4.0)	60	2.0 (0.0–3.5)	0.072
Number of intermediate/high-risk factors	110	3.0 (2.0–5.0)	24	4.0 (3.0–5.0)	26	3.0 (1.0–4.0)	60	4.0 (2.0–5.0)	0.25

Bold values denote statistical significance at the *p* < 0.05 level

cGGO, centrilobular ground glass opacifications; pGGO, panlobular ground glass opacification; IQR, interquartile range; RAPm, mean right atrial pressure; PAPs/d/m, systolic, diastolic and mean pulmonary artery pressures; CO, cardiac output; CI, cardiac index; SvO_2_, mixed venous blood oxygen saturation; PVR, pulmonary vascular resistance. *For comparison between three HRCT patterns

The abnormal HRCT pattern was more frequent in women (29% had pGGO, 25% cGGO, and 49% normal pattern) than in man (10% had pGGO, 13% cGGO, and 77% normal pattern), p = 0.004. Patients with abnormal pattern had lower median age: 37.5 (26.6–49.6) vs 46.8 (37.6–59.0) years, p = 0.029. The cGGO and pGGO groups had higher rates of patients with positive history of haemoptysis compared to the normal pattern group: 29% and 23% vs 9%, p = 0.046. There was no difference in WHO FC between the groups, but patients with the abnormal HRCT pattern had higher median value of 6MWD than patients with the normal pattern: 429 (380–484) vs 379 (298– 450) m, p = 0.018.

The distinctive features of the pGGO group were: (i) negative association with cigarette smoking: only 19% of these patients were ever-smokers vs 45% of the remaining patients, p = 0.019, (ii) higher number of low-risk factors: 3.0 (2.0–4.0) vs 2.0 (0.0–3.0) in the remaining patients, p = 0.024. The distinctive feature of the cGGO group was higher median RAPm: 10.0 (8.0–13.0) vs 7.0 (4.0–10.0) mmHg in the remaining patients, p = 0.008.

There were no significant differences between the lung HRCT patterns in terms of presence of patent foramen ovale, oxygen saturation, DLCO%pred, NT-proBNP, or haemodynamic parameters other than RAPm.

The results of logistic regressions are shown in Table 3. The independent predictors for pGGO were: higher number of low-risk factors, lower CO, and positive history of haemoptysis. The independent predictors for cGGO were: younger age, higher RAPm, higher Svo_2_, and positive history of haemoptysis.

**Table 3:** Univariate and multivariate logistic regression models for different patterns of ground glass opacification

	cGGO			
	Univariate		Multivariate	
	Odds ratio (95% CI)	p-value	Odds ratio (95% CI)	p-value
Haemoptysis	**4.36 (1.22–15.56)**	**0.023**	**6.34 (1.39–28.95)**	**0.015**
Age < 40 years	**4.22 (1.55–11.45)**	**0.005**	**4.56 (1.41–14.47)**	**0.01**
6MWD [m]	**1.005 (1.00007–1.01)**	**0.047**		
RAPm [mmHg]	**1.11 (1.005–1.23)**	**0.04**	**1.21 (1.05–1.38)**	**0.017**
Svo_2_ [%]	1.02 (0.97–1.08)	0.42	**1.11 (1.03–1.19)**	**0.01**

	pGGO			
	Univariate		Multivariate	
	Odds ratio (95% CI)	p-value	Odds ratio (95% CI)	p-value
Haemoptysis	3.18 (0.87–11.59)	0.08	**5.09 (1.04–24.96)**	**0.041**
Female gender	**4.77 (1.28–17.68)**	**0.02**		
Cigarette smoking	**0.28 ( 0.09–0.85)**	**0.024**		
Number of low-risk factors	**1.33 (1.02–1.73)**	**0.036**	**2.32 (1.46–3.69)**	**0.0003**
CO [l/min]	0.68 (0.43–1.09)	0.11	**0.31 (0.14–0.66)**	**0.002**

Bold values denote statistical significance at the *p* < 0.05 level

cGGO, centrilobular ground glass opacification; pGGO, panlobular ground glass opacification; 6MWD, 6-min walking test distance; RAPm, mean right atrial pressure; SvO_2_, mixed venous blood oxygen saturation; CO, cardiac output

### Survival

**Fig. 2:**
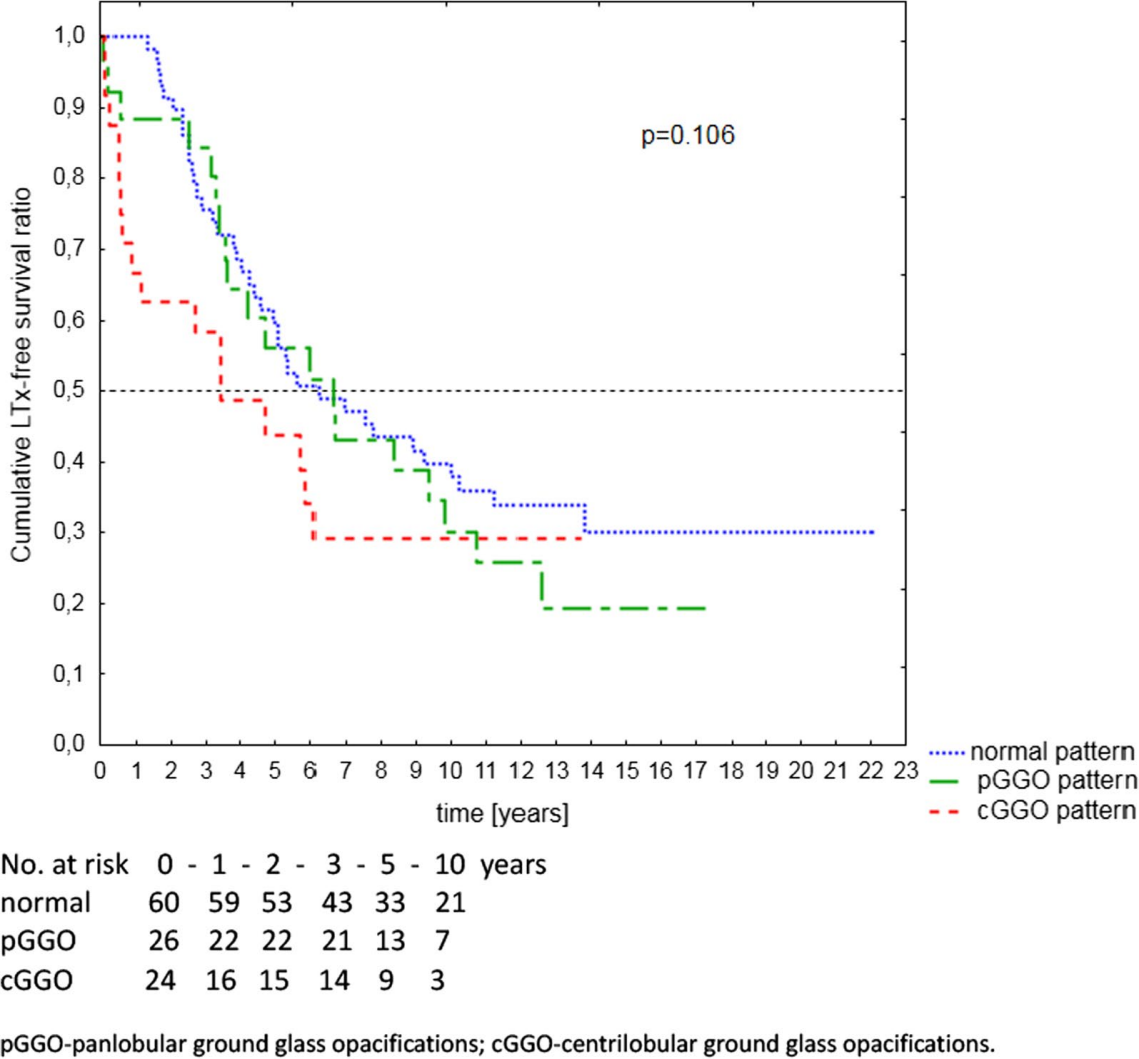
Kaplan–Meier curves showing LTx-free survival depending on the lung HRCT pattern

The median time of follow-up was 5.0 years, range 0.1–22.1 years. During this time 73 (66%) patients died (65 patients) or underwent LTx (8 patients), 26 (24%) patients were still alive at the end of the study, and 11 (10%) were lost to follow-up. The patients’ LTx-free survival depending on the lung HRCT pattern is illustrated in Fig. 2 and summarised in Table 4. The cGGO group had the lowest median LTx-free survival: 3.4 vs 6.2 and 5.8 years respectively for the pGGO and normal pattern groups. Within the first year occurred 50%, 15%, and 0% of all the deaths/LTx in the cGGO, pGGO and normal groups, respectively. 56% of unfavourable events in the cGGO group took place in first 2 years.

**Table 4:** Survival rates for the whole group and according to chest HRCT patterns

	Survival ratio (95%CI) [%]				p-value*
	Whole group	cGGG pattern	pGGO pattern	Normal pattern	
1-year	90.0 (84.4–95.6)	**66.7 (47.8–85.5)**	**88.5 (76.2–100.0)**	**100.0 (94.9–100.0)**	**0.00002**
2-year	84.4 (77.6–91.2)	**62.5 (43.1–81.9)**	**88.5 (76.2–100.0)**	**91.4 (84.2–98.6)**	**0.0006**
3-year	74.0 (65.7–82.3)	**58.3 (38.6–78.1)**	**84.4 (70.4–98.5)**	**75.4 (64.5–86.7)**	**0.024**
5-year	55.4 (45.9–64.9)	43.8 (23.2–64.3)	56.0 (365–75.6)	61.5 (48.9–74.6)	0.09
10-year	34.9 (25.5–44.2)	29.2 (9.9–48.4)	30.2 (11.7–48.6)	39.7 (26.9–52.6)	0.12

Bold values denote statistical significance at the *p* < 0.05 level

cGGO, centrilobular ground glass opacifications; pGGO, panlobular ground glass opacification

^*^For comparison between cGGO and joined two remaining patterns

For 71% of the patients all six risk factors were documented, further 24% had 5 risk factors available, 5% of patients had 2–4 risk factors documented. 27% of the patients were in the low-risk group, 60% in the intermediate-risk group, and 13% in the high-risk group. The median number of low-risk factors was 2, 23% of the patients had no low-risk factors, 27% had at least 4 low-risk factors. There were no significant differences in distribution of the HRCT patterns in the risk groups, but pGGO pattern was associated with a higher number of low-risk predictors compared to other patterns — Table 2. One- and two-year LTx-free survival didn’t differ significantly between the three risk groups (p = 0.3 and p = 0.5, respectively), or depending on number of low-risk factors (p = 0.8 and p = 0.4, respectively).

All three patients with cGGO and either lymphadenopathy or septal thickening had survival shorter than 1 year. The patient with pGGO, lymphadenopathy and septal thickening as well as all patients with normal pattern and lymphadenopathy survived longer than 2 years.

Significant independent predictors for negative outcome in the perspective of the whole follow-up were: intermediate/high risk group (HR 3.84, 95% CI 1.71–8.59) and DLCO%pred (HR 0.98, 95% CI 0.97–0.99). Independent predictors in the two-year perspective were: presence of cGGO (HR 6.22, 95 CI 1.37–28.18), DLCO%pred (HR 0.95, 95% CI 0.92–0.98), and decrease in oxygen saturation during 6-min walking test (HR 1.13, 95% CI 1.04–1.22). Results of uni- and multivariate Cox regressions are shown in Tables 5 and 6.

**Table 5:** Predictors of outcome in the 2-year perspective

	DEATH or LTX In a 2-year perspective			
	Univariate		Multivariate	
	Hazard ratio (95% CI)	p-value	Hazard ratio (95% CI)	p-value
WHO FC 4	**5.89 (1.64–21.19)**	**0.04**		
cGGO	**5.01 (1.95–13.00)**	**0.0009**	**6.22 (1.37–28.18)**	**0.018**
DLCO [%pred]	**0.96 (0.93–0.99)**	**0.001**	**0.95 (0.92–0.98)**	**0.005**
NT-proBNP [pg/ml]	**1.0003 (1.0001–1.0004)**	**0.003**		
6MWT ΔSpO_2_ [%point]	1.05 (0.999–1.1)	0.05	**1.13 (1.04–1.22)**	**0.004**

Bold values denote statistical significance at the *p* < 0.05 level

WHO FC, World Health Organisation functional class; cGGO, centrilobular ground glass opacifications; DLCO, diffusion lung capacity for carbon monoxide; NT-proBNP, N-terminal pro-brain natriuretic peptide; 6MWT ΔSpO_2_, change of blood oxygen saturation during 6-min walking test

**Table 6:** Predictors of outcome in the whole follow-up perspective

	DEATH or LTX in in the whole follow-up perspective			
	Univariate		Multivariate	
	Hazard ratio (95% CI)	P-value	Hazard ratio (95% CI)	P-value
Male gender	2.11 (1.29–3.45)	0.003		
Cigarette smoking	1.63 (1.01–2.64)	0.045		
WHO FC 3–4	1.92 (1.21–3.06)	0.006		
6MWD [m]	0.9977 ( 0.9957–0.9997)	0.026		
SpO_2_ [%]	0.91 (0.87–0.96)	0.0003		
6MWT ΔSpO_2_	1.04 (1.01–1.06)	0.009		
DLCO [%pred]	0.97 (0.96–0.99)	0.0001	0.98 (0.97–0.99)	0.002
NT-proBNP[pg/ml]	1.0002 (1.0001–1.0004)	0.0002		
RAPm [mmHg]	1.07 (1.02–1.12)	0.003		
CO [l/min]	0.77 (0.62–0.97)	0.026		
CI [l/min/m^2^]	0.59 (0.40–0.89)	0.01		
Svo_2_ [%]	0.97 (0.95–0.99)	0.013		
Non-responder	3.18 (1.51–6.69)	0.002		
Number of low-risk factors	0.76 (0.66–0.88)	0.0002		
Intermediate/high risk group	4.15 (2.05–8.37)	0.00007	3.84 (1.71–8.59)	0.001

WHO FC, World Health Organisation functional class; 6MWD, 6-min walking test distance; SpO_2_, blood oxygen saturation; 6MWT ΔSpO_2_, change of blood oxygen saturation during 6-min walking test; DLCO, diffusion lung capacity for carbon monoxide; NT-proBNP, N-terminal pro-brain natriuretic peptide; RAPm, mean right atrial pressure; CO, cardiac output; CI, cardiac index; SvO_2_, mixed venous blood oxygen saturation

## Discussion

Chest HRCT plays an essential role in the workup of PH [1], and it is also common that the study is repeated during the disease course for various clinical reasons. The number of previous reports on lung HRCT appearance in IPAH is very limited [3, 6, 11–17]. We present the largest and most thorough analysis of the lung parenchyma attenuation inhomogeneity on HRCT scans of IPAH patients so far. We found mosaic attenuation in 46% of our patients: pGGO in 24%, and cGGO in 22% of the patients. Rates were similar to previously reported [11, 12, 17] and confirmed high prevalence of GGO in these patients population. In only one study, including 15 IPAH patients, centrilobular pattern was more frequent than panlobular GGO pattern (80 vs 20%, respectively) [6].

In our study, the groups of patients with centri- and panlobular GGO patterns shared some similarities that distinguished them from the patients with normal pattern: more frequent female gender, younger age, better 6MWD, and positive history of haemoptysis. We failed to find explanation for association of GGO with gender and age. Better 6MWD could be secondary to younger age of these patients. Haemoptysis is a recognised complication of IPAH associated with poor prognosis [33]. The prevalence of haemoptysis in our study group was 16%. Tio et al. [34] reported haemoptysis in 4.8% of 228 I/HPAH patients, Montani et al. [3] in 8.3% of 24 I/H/anorexigen-induced PAH, Ghigna et al. [35] in 57% of 44 patients who underwent LTx for I/HPAH. In the group reported by Tio et al. [34], patients with haemoptysis in anamnesis were younger, had earlier PH onset and more dynamic haemodynamic worsening than remaining patients. Data on lung imaging were not provided. Haemoptysis in PAH results from concomitant involvement of bronchial circulation and intrapulmonary bronchopulmonary anastomoses leading to pulmonary haemorrhage [36]. Alveolar haemorrhage is one of the explanations of GGO presence in PAH patients. Depending on bleeding extend and phase, it may appear either as cGGO or pGGO (patchy or confluent) [37]. In about 25% of patients with various forms of PH, cholesterol granulomas with siderophages could be found in lung specimens [15, 38], and they were thought to be secondary to haemorrhage. There is no consensus whether cGGO seen on lung imaging studies correspond to cholesterol granulomas, small foci of haemorrhages, or extensive plexogenic arterial lesions [15, 38, 39].

We found features distinctive for each GGO pattern group. The cGGO group was characterised by the highest RAPm, and that was in agreement with previous studies on smaller groups [11, 17]. Independent predictors of cGGO were: younger age, positive history of haemoptysis, higher RAPm, and higher Svo_2_. The pGGO group had the lowest rate of cigarette smokers and the highest median number of low-risk factors. Independent predictors of pGGO were: positive history of haemoptysis, higher number of low-risk factors, and lower CO.

We also demonstrated significant differences in short-term survival rates between the groups. Presence of cGGO turned out to have a negative prognostic value for outcome in a 2–year perspective, with sixfold increase in death/LTx risk. The 1,2,3-year survival rates in the cGGO group were similar to those reported for high-risk group PAH patients in national and international registries [20, 22], although only 17% of cGGO patients were in the high-risk group at the baseline assessment. No prognostic value of pGGO compared to normal pattern was demonstrated. In our group, the prognostic value of HRCT pattern was superior to ESC/ERS risk stratification approach in respect to a 2-year survival, but it lost its significance in favour of the latter in the long-term perspective. This could be explained by the high mortality in the cGGO group in the first years of follow-up, and small number of these patients staying in observation for longer than 6 years. It is also worth noting the significant prognostic value of DLCO%pred both in short and long observation.

In the clinical context of PAH with GGO present on HRCT lung scans, it is of utmost importance to differentiate between IPAH and PAH with overt features of venous/capillaries (PVOD/PCH) involvement [1]. Survival in PVOD/PCH is significantly worse than in other forms of PAH, with reported mean LTx-free survival 1–2.5 years [3, 4], and treatment with pulmonary vasodilators can cause pulmonary oedema [1]. A suspicion of PVOD/PCH is an indication for LTx referral [1, 27, 28]. Although ultimate distinction can be made only on histopathologic or genetic basis, it is believed that diagnosis of PVOD/PCH may be established with high probability using a combination of non-invasive tests [1, 3, 5, 6]. Diagnostic clues include very low DLCO, resting hypoxemia, severe exertional desaturation, and characteristic features on chest HRCT [1, 3, 5]. A triad of centrilobular ground-glass opacities, mediastinal lymphadenopathy, and smooth thickening of the interlobular septa is very suggestive for PVOD/PCH. Presence of at least two of the three features has a sensitivity of 75% and specificity of 85% for PVOD/PCH; normal chest HRCT doesn’t exclude PVOD/PCH, though [3, 5]. It’s estimated that 3–12% of IPAH clinical diagnoses are indeed PVOD/PCH [5]. It is possible that some patients in our cohort could have had PVOD/PCH. The radiological triad was seen in none of our patients, two patients with cGGO had lymphadenopathy, another one had septal thickening, one patient with pGGO had lymphadenopathy and septal thickening. According to the HRCT picture, 3.6% of our patients had higher level of suspicion of PVOD/PCH. On the other hand, in patients with I/H/anorexigen-induced PAH, with no signs of PVOD/PCH on lung pathologic evaluation, cGGO were seen in 23–28%, septal thickening in 13–15%, and lymphadenopathy in 8% [3, 6, 15], and coexistence of at least two of these findings was present in 15% of the patients [3]; so specificity of cGGO for PVOD was only 73–77% [3, 6]. As lung biopsy is contraindicated in PH, and access to genetic testing is limited, many PVOD/PCH diagnoses in reality are based on clinical observation. Applying a proper management is more important that making the specific diagnosis. General principles of referring patients for LTx are: (i) a 2-year predicted survival of < 50%, and (ii) a high likelihood of post-transplant survival [27]. The results of our analysis indicate, that in patients with clinical diagnosis of IPAH with cGGO on the lung HRCT scans an early referral to LTx centres should be considered. With such approach, evaluation of the disease dynamic and response to medical treatment on one hand, and necessary LTx-related assessment on the other hand, could be done in parallel, improving timing for LTx listing.

Our research has shortcomings related to its retrospective character, possible selection bias, disregard of medical treatment in the analysis, and still small number of included patients. Nevertheless, it gives insights into different phenotypes of IPAH patients depending on lung HRCT pattern and proves independent significance of cGGO in predicting survival in a real-life patients cohort.

Further research is needed to elucidate many intriguing issues related to IPAH phenotype associated with GGO on lung HRCT, e.g. the histopathologic background of cGGO and pGGO, mutual dependence of causes and effects between GGO-related structures and PH course, relation between GGO and right ventricle performance assessed with echocardiography and/or cardiac magnetic resonance imaging, or effect of PAH-specific treatment on GGO.

## Conclusions

GGO are frequent findings on the lung HRCT scans of IPAH patients. Panlobular and centrilobular patterns of GGO are equally prevalent, but have different clinical significance. Presence of cGGO on HRCT scans of the lungs of IPAH patients has an add-on prognostic value for an outcome in a 2-year follow-up in reference to already well-established prognostic factors collected in the ESC/ERS multidimensional risk assessment tool, and could serve as an complementary element in making therapeutic decisions, e.g. the timing of referral for LTx assessment.

## Data Availability

The datasets used and analysed during the current study are available from the corresponding author on reasonable request.

## Abbreviations

PH: Pulmonary hypertension
PAPs/d/m: Systolic, diastolic, mean pulmonary artery pressures
RHC: Right heart catheterisation
PAH: Pulmonary arterial hypertension
PVR: Pulmonary vascular resistance
PAWP: Pulmonary artery wedge pressure
IPAH: Idiopathic pulmonary arterial hypertension
HPAH: Heritable pulmonary arterial hypertension
HIV: Human immunodeficiency virus
BMPR2: Bone morphogenetic protein receptor 2
PVOD: Pulmonary veno-occlusive disease
PCH: Pulmonary capillary haemangiomatosis
LTx: Lung transplantation
EIF1AK4: Eukaryotic translation initiation factor 2alpha kinase 4
DLCO: Lung diffusion capacity for carbon monoxide
HRCT: High resolution computed tomography
GGO: Ground-glass opacities
cGGO: Centrilobular ground-glass opacities
pGGO: Panlobular ground-glass opacities
ESC: European Society of Cardiology
ERS: European Respiratory Society
WHO FC: World Health Organisation functional class
NT-proBNP: N-terminal pro-brain natriuretic peptide
%pred: Percent of predicted value
RAPm: Mean right atrial pressure
CO: Cardiac output
CI: Cardiac index
Svo_2_: Mixed venous blood oxygen saturation
IQR: Interquartile range
6MWD: Six-minute walking test distance
HR: Hazard ratio
95% CI: 95% Confidence interval
PFO: Persistent foramen ovale
SpO_2_: Blood oxygen saturation
